# Familial risks in prostate cancer between brothers and half-brothers as clues to germline genetic and environmental causes

**DOI:** 10.1007/s10689-026-00562-3

**Published:** 2026-05-11

**Authors:** Kari Hemminki, Frantisek Zitricky, Kristina Sundquist, Jan Sundquist, Asta Försti, Akseli Hemminki, Otto Hemminki

**Affiliations:** 1 Faculty of Medicine, Biomedical Center, Charles University, 30605 Pilsen, Czech Republic; 2 Division of Cancer Epidemiology, German Cancer Research Center (DKFZ), Im Neuenheimer Feld 580, 69120 Heidelberg, Germany; 3 Center for Primary Health Care Research, Lund University, 205 02 Malmö, Sweden; 4 University Clinic Primary Care Skåne, Malmö, Sweden; 5 Department of Family and Community Medicine, McGovern Medical School, The University of Texas Health Science Center, Houston, TX USA; 6 Hopp Children’s Cancer Center (KiTZ), Heidelberg, Germany; 7 Division of Pediatric Neurooncology, German Cancer Research Center (DKFZ), German Cancer Consortium (DKTK), Heidelberg, Germany; 8 Comprehensive Cancer Center, Helsinki University Hospital, Helsinki, Finland; 9 Translational Immunology Research Program, Cancer Gene Therapy Group, University of Helsinki, Helsinki, Finland; 10 Department of Urology, Helsinki University Hospital, Helsinki, Finland

**Keywords:** Familial risk, Germline genetics, Heredity, Half-brother, Age of onset

## Abstract

Swedish family and cancer data constitute the largest source on familial cancer in the world. We analyze here familial risks in prostate cancer (PC) with focus on multiple affected brothers and comparation of full-brothers to maternal and paternal half-brothers. Age-specific incidence and standardized incidence ratios (SIRs) were calculated for PC in brothers. Curves for relative risk (RR) by diagnostic age were plotted for risk distributions. A total of 115,066 PCs were diagnosed in 1.2 million men. Familial SIR for full brothers was 2.23, for maternal half-brothers it was 1.92 and paternal half-brothers was 1.34. Considering SIRs with least possible detection bias (7+ years after first brother’s diagnosis) the above SIRs were 2.06, 1.66 and 1.41. SIRs in full brothers increased stepwise by the number of affected brothers reaching 21.33 when 6 brothers were affected. Age-RR curves for two affected brothers declined evenly from RR 2.8 at age 45 to below 2.0 at age 80. When four or more brothers were affected, a discrete high-risk peak (RR 4–7) was detected between ages 60 and 69. Data on full-brothers and half-brothers indicate that familial risk in PC is largely genetic which is also supported by discrete RR peaks in high-risk families at ages matching preferential penetrance age for known predisposition genes of PC. Familial risk increased already when two brothers were affected calling for clinical vigilance concerning family history. Family history should deserve a place as an inclusion criterium in schemes for PC screening.

**Supplementary Information:**

The online version contains supplementary material available at 10.1007/s10689-026-00562-3.

## Introduction

Familial clustering of cancer was initially observed for rare cancers in families with many affected individuals, later recognized as retinoblastoma and Li-Fraumeni syndromes [1]. However, familial cancer gained a wider population-level impact through the collection of colorectal cancer families in the 1960s and 1970s by Henry Lynch, leading to the detection of the mismatch repair gene mutations as the underlying cause [2, 3]. Parallelly, gene finding efforts in breast cancer families led to the identification of the genes *BRCA1* and *BRCA2* [1]. These discoveries boosted efforts for identification of novel cancer predisposition genes in families with an apparent clustering of certain cancers (linkage studies) and many new genes were discovered until about year 2002 [1]. For prostate cancer (PC), linkage studies have not been helpful and for the current PC predisposition genes (*BRCA2*, *HOXB13*, *BRCA1*, *CHEK2*, *PALB2, ATM2* and mismatch repair genes) the verification has been done in case–control studies between large numbers of patients whose germline DNA has been sequenced [4–6]. Combined pathogenic germline variants in the above genes may account, according to a review, for 3 to 5% of localized PC, 7% of familial PC and 12% of metastatic PC [7]. The increasing variant proportion towards familial and metastatic patients suggests that such cases are enriched in variants with an aggressive phenotype [6, 8–10]. In Scandinavia, PC patients frequently harbor *HOXB13* mutations which were not included in the studies reviewed above [7, 9, 11].

In addition to the above genes, low-penetrance variants contribute to familial risk of PC, and risks combined for 100 or more loci may be calculated as the polygenic risk score (PRS) [12–14]. Familial clustering and PRS are correlated, but independent; principally the genes/variants contributing to familial risk and PRS differ by the frequency of variants in the population (higher for PRS) and effect size (higher for predisposition genes) [15]. The risks between these two types of variants are additive and may include also life-style risk factors [16]. In general, the distinction between genetic and environmental effects may be difficult in a familial setting, and e.g., familial risk between spouses (correlation of cancer risk between spouses) has been one way to estimate environmental effects [17]. Another powerful option is to compare cancer risk between full- and half-siblings for whom data are uniquely available in Sweden [18].

We apply here the nationwide updated family data of Sweden covering essentially a century with cancers from 1958 recorded in the national high-quality cancer registry [19]. The special feature of the family data is their completeness for 16 million individuals, also including half-siblings have been recorded after parental divorce. In Sweden, children normally remained with the mother until about 1990 when joint custody of children became more common [20]. The earlier versions of the dataset have been used in several family studies on PC [21–24]. Using this resource we focus on age-specific analysis of familial PC between brothers (i.e., a single generation) based on the number of affected brothers, which age-specific data should be informative of the penetrance age of high-risk germline variants. Familial risk between brothers was selected because the difference in their diagnostic ages was smaller than between fathers and sons, which was considered important in view of the changing epidemiology of PC (fathers were diagnosed largely before the prostate specific antigen (PSA) era starting at around 1990 [25]). The main focus is on full-brothers (also ‘brothers’) and half-brothers. In further analyses, family members with selected discordant cancers of the breast and colorectum were included in attempts to delineate genetic effects by genes shared by PC and these other cancers. Previously we have shown the usefulness of the approach on various cancers without specifying family history [26, 27]. Any reliable risk factors for PC are urgently needed because of the controversies about ongoing and planned screening practices [28].

## Methods

The analyzed dataset originates from the latest update of the Swedish Cancer Registry (covering period 1961–2021), linked with the Multigeneration Register, which includes the Swedish population: generation 1 born before 1932 (median birth year 1912), generation 2 born in Sweden from 1932 onwards (median birth year 1947) and generation 3 children of parents from generation 2 (median birth year 1976). The present study focused on men in generation 2, except that breast and colorectal cancers were obtained for all first-degree relatives (FDRs). The oldest persons in generation 2 reached age 89 years by 2021. Persons born after 1932 with missing links to parents were excluded. Half-brothers were available in the database, for maternal ones with the same biological mother and different father; for paternal ones, with the same biological father and different mother [18]. All family and cancer linkages were done through the unique personal identification number. Cancers at specific sites were diagnosed through the ICD-7 codes for PC (177), breast cancer (BC, 170) and colorectal cancer (CRC, 153 and 154).

Incidence rates were calculated in 5-year age-brackets for incident cases in a defined age-group divided by person-years at risk in the same age-group. Cases were the sets of brothers who were diagnosed with PC. Reference rates were calculated for men without FDR diagnosed with PC. Rate ratios (RRs) were calculated by dividing the case incidence rate by the reference incidence rate, and the age-RR curves are shown with the incidence curves. Age-specific incidence and RRs were also developed for brothers diagnosed with PC when family members were diagnosed with discordant cancers of the breast and colorectum. The incidence rates were also calculated for specified time interval with respect to sibling’s diagnosis date. The incidence rates for first diagnosed brother were calculated by considering that he and any of his brothers were at risk only until his (first brother’s) diagnosis date.

**Fig. 1:**
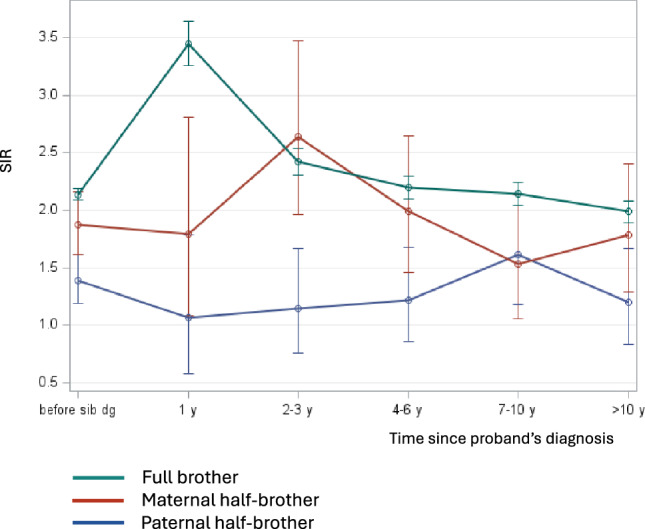
Familial risks (SIRs and 95% CIs) for PC for the second brother since diagnosis of the first brother at time 0, considering full brothers and maternal and paternal half-brothers

In analysis of familial relative risk, one person of each family in generation 2 was defined as case and his full brothers or half-brothers diagnosed with PC were defined as probands. The calculation was based on the standardized incidence ratio (SIR) as the ratio of the observed number of PCs in population at risk to the expected number of PCs, i.e., cases whose FDR and half-brothers were not diagnosed with PC. The follow-up started on date of birth or beginning of the study (1st January 1961), whichever came later. The follow-up was terminated at time of death, emigration, PC diagnosis or end of study (29th December 2021), whichever came earliest. In SIR estimation with respect to brother’s diagnostic date (Fig. 1 and SIR for first brother with PC in Table 1), the follow-up was further restricted to the defined time period. The rates were standardized based on sex, age (5-year groups), calendar period (10-year groups), educational level (< 9 years, 9 years, 10–11 years, 12 years, college < 3 years, university graduate, postgraduate) and geographic region (north, south and 3 largest cities). The 95% confidence intervals (CIs) were calculated assuming that observed rates follow Poisson distribution.

**Table 1:** Relative risk (SIR) for PC based on brothers diagnosed with PC (full brothers, maternal and paternal half-brothers) with cancer in any brothers or in first brother only

Brothers with PC			Risk to any brother		Risk to first brother	
Full brother	Maternal HB	Paternal HB	N familial	SIR [95% CL]	N familial	SIR [95% CL]
0	0	2	339	1.34 [1.20–1.49]	178	1.39 [1.20–1.61]
0	2	0	382	1.92 [1.73–2.12]	188	1.87 [1.61–2.16]
0	2	2	16	2.75 [1.57–4.46]	7	2.74 [1.10–5.66]
2	0	0	16,290	2.23 [2.19–2.26]	8139	2.14 [2.09–2.19]
3	0	0	2429	3.59 [3.45–3.74]	806	3.34 [3.12–3.58]
4	0	0	398	5.09 [4.60–5.62]	98	4.25 [3.45–5.18]
4	0	0	84	6.94 [5.53–8.59]	16	5.40 [3.09–8.77]
6	0	0	53	21.33 [15.98–27.9]	9	16.78 [7.68–31.87]

N = number of observed familial PC cases in respective family constellations (see methods). Fist brother is the first one diagnosed in a brothership

Throughout the manuscript, groups with estimated SIRs were labeled by total number of PC patients that would result if the followed person (case) was diagnosed with PC. Therefore, “2 brothers with PC” designates risk estimates for men with one brother proband diagnosed with PC.

All statistical analyses and data visualization were done using SAS and R (version 4.4.0).

Data on penetrance age for predisposition genes are presented in Supplementary Table 1.

## Results

Number of men in generation 2 (1,171,247), number with diagnosed PC (115,070; 9.5%).

Families with a single PC in generation 2 accounted for 83.2% of all, those with families of 2, 3 and ≥ 4, PCs accounted for 14.2, 2.1 and 0.5%, respectively.

### Familial risks for full and half-brothers

Case numbers and SIRs are shown in Table 1**,** separately considering risks for PC in all brothers in families with multiple brothers or in such families for the first diagnosed brother only. The reason for the two types of analyses was that the diagnosis in the first brother would not be biased by diagnosis in other brothers. In the case of half-brothers there was no difference between the SIRs. In column ‘risk to any brother’ SIR for paternal half-brothers was 1.34 and that for maternal half-brothers was 1.92 with a significant difference. SIR for full brothers was 2.23, significantly higher than that for either half-brothers.

Comparing SIRs for full brothers we can note that risk increases according to the number of affected brothers (Table 1). SIR reached 21.33 for 6 affected brothers (column ‘risk to any brother’) but only 16.78 when risk to the first brother was considered. The difference in SIRs between two columns increased by the number of affected brothers.

According to the literature there is diagnostics bias for PC between brothers, such that the diagnosis of the first brother may cause concerns and facilitation of medical contacts in unaffected brothers [29, 30]. We thus determined familial risks for brothers at various time intervals since diagnosis of the first brother (Fig. 1). Full-brothers diagnosed within a year showed the highest SIR of 3.4, and a steeply decreasing risk reaching 2.0 in > 10 years. For maternal half-brothers the peak SIR of 2.7 was reached at 2–3 years, followed by a decline to 1.7. For paternal half-brothers a modest increase in risk to 1.6 was noted at 7–10 years. The median difference in diagnostic time between the first and the second brother was 58 months for full-brothers, 55 months for maternal half-brothers and 74 months for paternal half-brothers.

According to Fig. 1 it seemed that the risks between brothers stabilized with extended time difference between the diagnoses of the brother pairs. Considering also case numbers, we selected the period past 7 years of first brother’s diagnosis for the least biased comparison. The SIR after 7+ years for full-brothers was 2.06 (N 3423, 95% CI 1.99–2.13); for maternal half-brothers it was 1.66 (N 77, 95% CI 1.31–2.08) and for paternal half-brothers it was 1.41 (N 82, 95% CI 1.12–1.74).

### Familial age-specific risks for PC

**Fig. 2:**
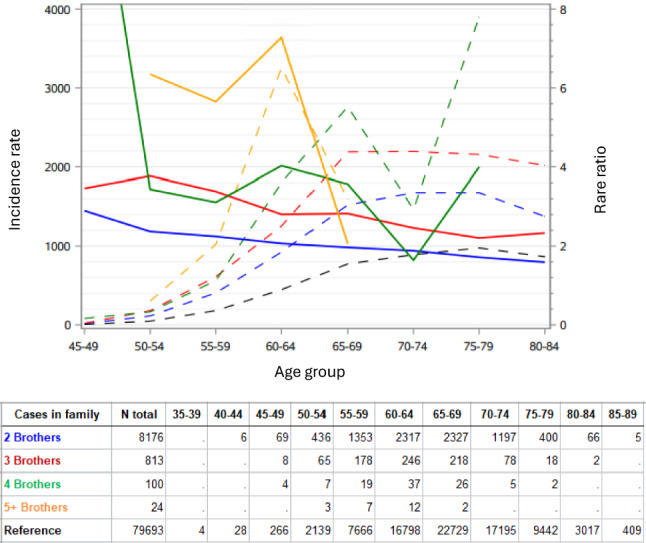
Age-specific incidence (left y-axis, dashed lines) and rate ratio (RR, right y-axis, solid lines) for the first diagnosed PC depending on the number of affected brothers in the family. The color code shows the number of affected brothers. The bottom table shows the case numbers in each data point

Age-specific RRs for PC in the first diagnosed brother depending on the number of affected brothers (irrespectively of PC in other first-degree relatives) are shown in Fig. 2 (solid lines). The corresponding incidence curves are shown by broken lines and case numbers at each RR data point are shown below the figure. The age-RR for 2 affected brothers decreased linearly from RR of 2.8 to RR 1.8. The RR for 3 affected brothers similarly declined, with two modest upward bends, from 3.3 to 2.2. The high RR for 4 brothers in age group 45–49 (N = 4) decreased rapidly and peaked again at age 60–64 years (RR 4, N = 37) extending to 65–69 years (N = 26). For 5+ affected brothers RR reached a peak of RR = 7 at age 60–64 years (N = 12).

### Likely contribution of known predisposition genes

In order to suggest origins for the discrete peaks between ages 50 and 70 years in Fig. 2 we consulted Supplementary Table 1 which listed the median penetrance ages for *BRCA1* and *BRCA2* at 66 and 71 years. Those for *MSH2* and *MLH1* were 65 and 70 years. We thus searched Swedish PC families additionally presenting with familial breast cancer (BC, for *BRCA* genes) or with familial colorectal cancer (CRC, for mismatch repair genes).

**Fig. 3:**
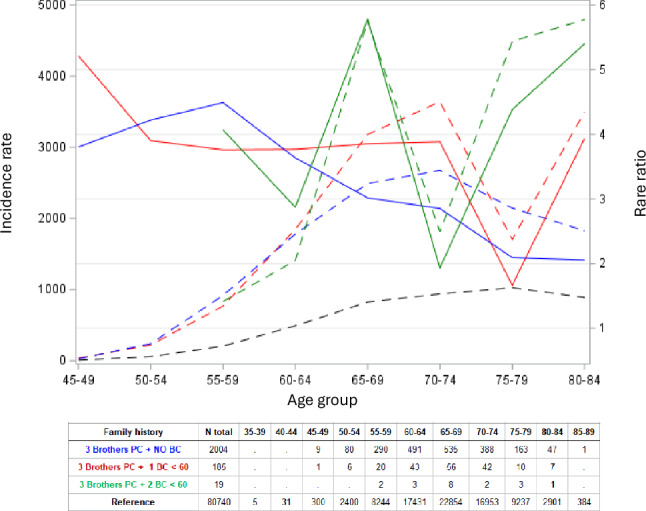
Age-specific incidence (left y-axis, dashed lines) and rate ratio (RR, right y-axis, solid lines) for PC when three brothers were diagnosed with PC regardless of cancers in FDR (blue lines); additionally, one FDR was diagnosed with BC before age 60 years (red lines) or two FDRs were diagnosed with BC before age 60 years (green lines)

Age-RR curves for the PC/BC families are shown in Fig. 3 when three brothers were diagnosed with PC. The blue curve depicts age-RR when female family members had no BC before age 60 years; red curve depicts families with one and green curve families with two BCs diagnosed before age 60 years, showing a solid peak with an RR of over 5 (N = 8) at age 65–69 years. However, as the case numbers were limited we relaxed the condition for PC to include only two affected brothers Supplementary Fig. 1. The green curve depicts families with additionally two BC diagnosed before age 60 years also show a peak (N = 22) at age 65–69 years.

**Fig. 4:**
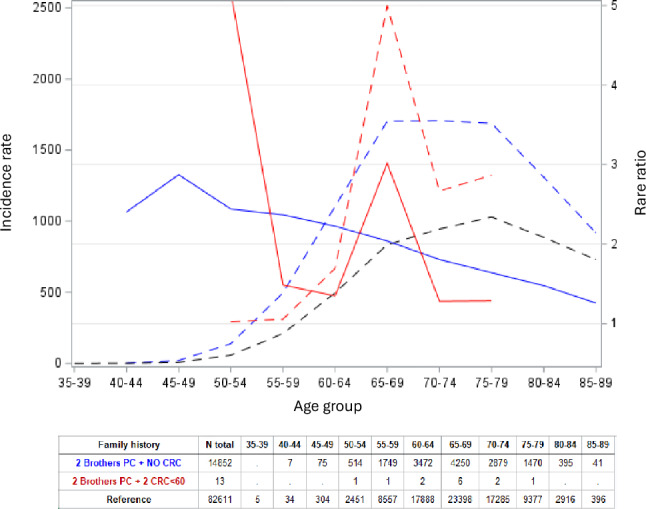
Age-specific incidence (left y-axis, dashed lines) and rate ratio (RR, right y-axis, solid lines) for PC when three brothers were diagnosed with PC (blue lines) and additionally two FDRs were diagnosed with CRC before age 60 years (red lines)

Similar data for familial PC are shown in Fig. 4 when there were family members diagnosed with CRC before age 60 years. Because of low case numbers, only PC families of affected brother pairs could be presented. In Fig. 4 in families with two CRCs a sharp peak of RR over 3.0 (N = 6) was recognized at age 65–69 years. Because of the low case numbers we reanalyzed the data with only one family member diagnosed with CRC before age 60 years (Supplementary Fig. 2). A broad increase in age-RRs covered age groups 65–69 to 80–84 years with most cases at age 65–69 years (N = 157).

## Discussion

The present study highlights three novel aspects of familial PC. Firstly, we showed distinct risks for full brothers and half-brothers which varied by the time interval between diagnosis of the two brothers. For full brothers the highest SIR of 3.4 followed immediately (within the same year) the diagnosis of the first brother. For maternal half-brothers the peak SIR of 2.7 was reached at 2–3 years and for paternal half-brothers a modest peak of 1.6 was noted at 7–10 years. The second novel result was the demonstration of vastly increasing risk and different age-RR relationships between families with increasing numbers of affected brothers, most likely signaling variation in genetic background. Age-RR curves in such families pinpointed age-groups of highest risk. The third novelty was the demonstration of the limits for large family studies on PCs to pinpoint discrete genes. We discuss the implications of these findings below.

Half-brothers share 25% of their genes compared to 50% sharing for full-brothers, and environmental sharing is expected to be much higher for maternal than paternal half-brothers because of maternal custody (see Introduction). Theoretically, the difference in cancer risk between maternal and paternal half-brothers would indicate contribution of environmental sharing. However, medical intervention (if maternal half-brothers are more likely to seek medical contact than paternal half-brothers) may distort the theory as was quite pervasively shown in Fig. 1. The peak risk was reached fastest for full brothers (within the first year), then for maternal half-brothers (2–3 years) and latest for paternal half-brothers (7–10 years). The median diagnostic time difference between brothers was largest for paternal half-brothers (74 months), deviating from the others (58 and 55 years, respectively), which may be related to sparse contacts between paternal half-brothers. We suggested that the most objective assessment (with least detection bias) of sibling risks would be the period 7+ years after diagnosis of the first brother; the SIRs were 2.06 for full brothers, 1.66 for maternal and 1.41 for paternal half-brothers. Case numbers for half-brothers were few but their risks were significantly increased over SIR 1. The combined risk for half-brothers (1.54, extra risk 1.54–1.00 = 0.54) was exactly 50% of that for full brothers (2.06, extra risk 1.06). However, the small difference between maternal and paternal half-brothers (1.66 vs. 1.41) suggests some environmental contribution to the risk of PC.

Cancers with a hereditary component tend to show many affected individuals and thus high familial risk. Here for full-brothers familial risk increased from 2.23 (two brothers with PC) to 3.59, 5.09, 6.94 and 21.33 (up to 6 brothers). Hereditary cancer is often diagnosed at a relatively early age compared to sporadic or life-style related cancers [31]. The basis for this is the multi-stage model of carcinogenesis whereby in hereditary cancer one or more genes are constitutionally mutated and thus bypassed already at birth leading to earlier onset of cancer [32]. Many familial cancers are also of relatively early onset but there are others with environmental contribution that appear at an advanced age [33]. The age-RR curves may transmit information about the underlying genes but the odds of detecting clear peaks of predisposition genes may not be high in view of the literature review which stated that combined known pathogenic germline variants account for no more than 7% of familial PC [7]. With the modest familial risk of two brothers we observed an almost linear declining RR-curve (Fig. 2), resembling the age-dependent declining risk by PRS of low-risk genes for which rare predisposing genes could contribute [14]. At higher number of affected brothers some discrete peaks appeared: with 4 brothers a broad peak with many patients emerged at age 60–69 years which for 5 + affected brothers narrowed to age of 60–64 y reaching an RR of 7. Among the known genes predisposing to PC at a preferential age range of 60–64 years include *ATM, CHEK2, HOXB1*3 and *BRCA1* (including also Swedish PC patients, Supplementary Table 1). The preferential age for *MSH2, MLH1*and *BRCA2* is on the higher side of that age range. However, a note of caution relating to the high risk of PC for the subsequent brothers after first brother’s diagnosis is that some detection bias may be present as pointed out in relation to Table 1.

We attempted also more specific genetic assignments for *BRCA1* and *BRCA2* and mismatch repair genes. We thus incorporated BC patients diagnosed before age 60 years into PC families and showed a discrete peak at age 65–69 years in line with predisposition caused by *BRCA1* and *BRCA2* (Fig. 3). Similar data for familial PC incorporating family members diagnosed with CRC before age 60 years defined an excess RR at age 65–74 years (Fig. 4), the range known for mismatch repair gene mutations in PC patients (Supplementary Table 1). The sizes of the observed peaks were small which should be considered against the background that the prevalence of any known predisposition genes for PC is much less than 2%, [4, 8, 9]. Thus, the prerequisite for seeing any discrete peaks would be a strong enrichment of familial cases which we achieved here. However, because of many age truncations it would not be possible to assess true numbers of mutation carriers, and no Swedish genetic reference data are available.

The basic limitation in this kind of study is the small numbers of familial cases in a North European country of 10 million people. However, during and after the Second World War a large number (1.8 million) of immigrants arrived in Sweden, initially from Europa but later also from other continents [34]. In non-European immigrants PC incidence was 0.45 and survival 0.6 compared to the Swedish level; incidence and survival in European immigrants was closer to the natives [35]. Comparison of familial risks for PC in Sweden to those in Iceland or Utah shows large similarities [36, 37].

In conclusion, this largest family study on PC yet conducted suggested that, in addition to the main genetic component, a minor environmental component, revealed in analysis of half-brother, may contribute to the familial risk of PC. Familial risk increased step-wise by the number of affected brothers reaching a clinically alarming SIR of 21.33 (6 brothers in a total of 53 families). Age-RR analysis of two-brother families revealed a broad age distribution of risk, probably contributed by a combination of low-risk polygenes combined with environmental factors. When 4 or more brothers were affected, a discrete high-risk age range covered years 60–69 which is the preferential age range of the know predisposition genes for PC. The results illustrate a ‘low risk-high risk’ heterogeneity of familial risk for PC which should be considered in future etiological studies. There are no generally accepted screening methods for PC, and The European Association of Urology recommends risk adapted strategies for well-informed men interested in a tailored approach for finding PC [38]. Certainly, positive family history among FDRs is a risk factor, yet the present study lacking paternal data and information of second-degree relatives is limited for detailed clinical risk prediction. Unfortunately, many ongoing PSA-based PC screening trials do not consider the readily available family history. The present results emphasize the need to take family histories for newly diagnosed PC cases and to consider referral to genetic counseling if two or more FDRs have been diagnosed with PC.

## Supplementary Information

Below is the link to the electronic supplementary material.

Supplementary file1 (DOCX 82 KB)

Supplementary file2 (DOCX 83 KB)

## Data Availability

Anyone wishing to use these data should contact the National Board of Health and Welfare, Stockholm.

## Abbreviations

BC: Breast cancer
FDR: First-degree relative
CRC: Colorectal cancer
PC: Prostate cancer
PRS: Polygenic risk score
PSA: Prostate specific antigen
RR: Relative risk
SIR: Standardized incidence ratio
