# Supplementary material for: Familial risks in prostate cancer between brothers and half-brothers as clues to germline genetic and environmental causes
Source: Fam Cancer. 2026 May 11;25(2):51. doi: 10.1007/s10689-026-00562-3 (PMC13161322; doi:10.1007/s10689-026-00562-3)
Supplement: Supplementary file 1 — Supplementary file1 (DOCX 82 KB) [file 10689_2026_562_MOESM1_ESM.pptx]

## Slide 1
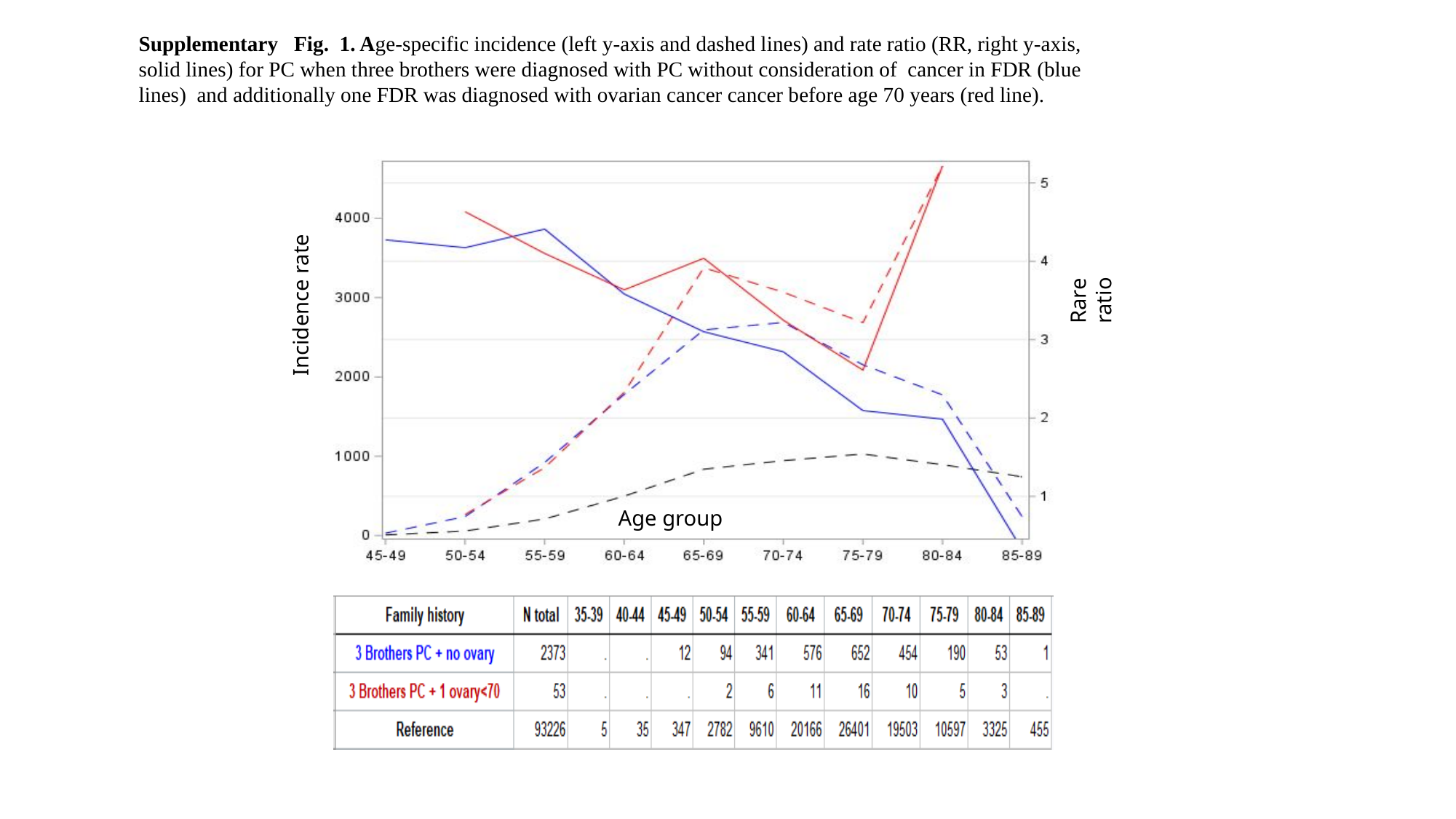

Supplementary Fig. 1. Age-specific incidence (left y-axis and dashed lines) and rate ratio (RR, right y-axis, solid lines) for PC when three brothers were diagnosed with PC without consideration of cancer in FDR (blue lines) and additionally one FDR was diagnosed with ovarian cancer cancer before age 70 years (red line).
Rare ratio
Incidence rate
Age group

## Slide 2
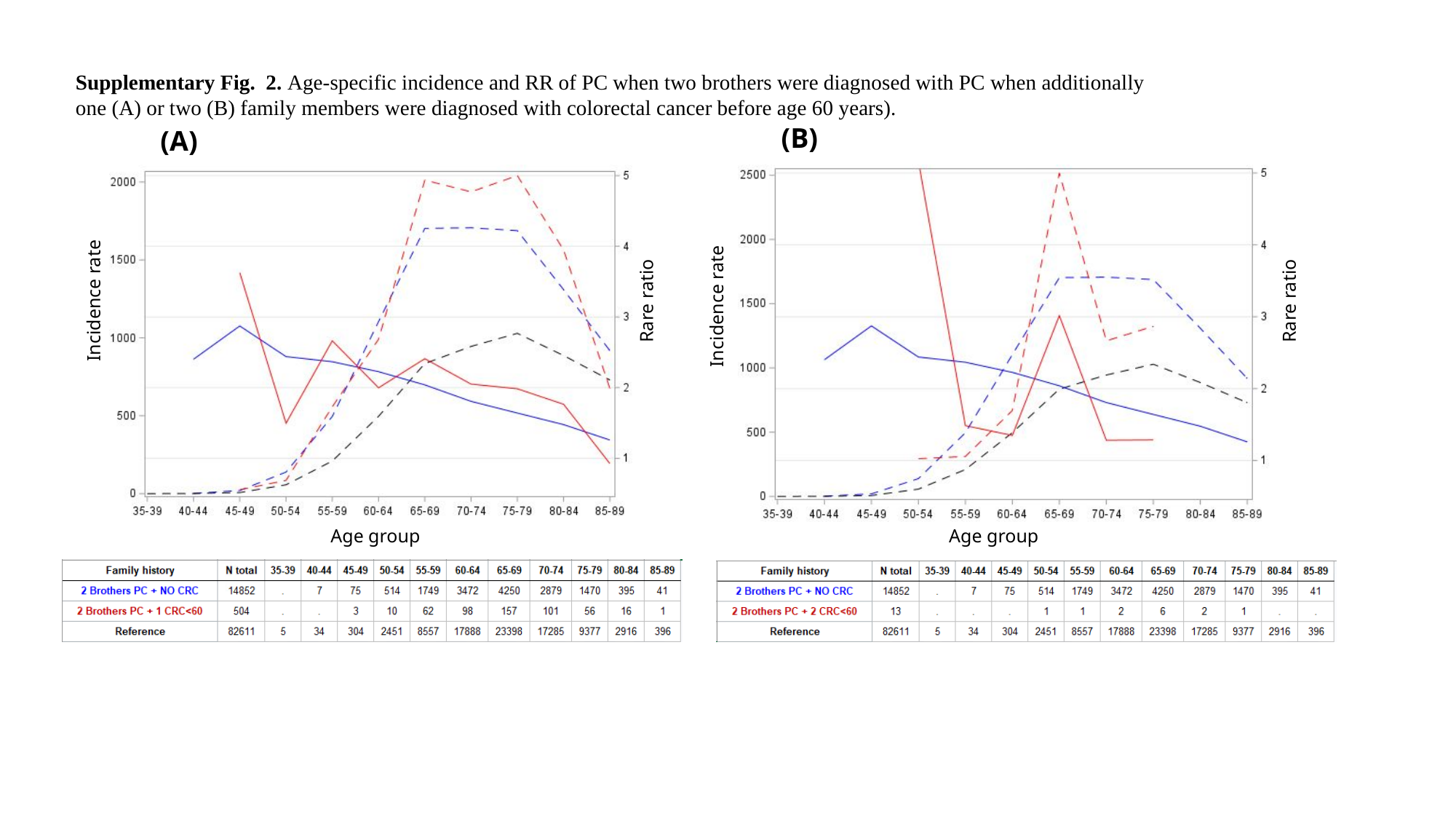

Supplementary Fig. 2. Age-specific incidence and RR of PC when two brothers were diagnosed with PC when additionally
one (A) or two (B) family members were diagnosed with colorectal cancer before age 60 years).
(B)
(A)
Rare ratio
Rare ratio
Incidence rate
Incidence rate
Age group
Age group
