# Supplementary material for: Familial risks in prostate cancer between brothers and half-brothers as clues to germline genetic and environmental causes
Source: Fam Cancer. 2026 May 11;25(2):51. doi: 10.1007/s10689-026-00562-3 (PMC13161322; doi:10.1007/s10689-026-00562-3)
Supplement: Supplementary file 2 — Supplementary file2 (DOCX 83 KB) [file 10689_2026_562_MOESM2_ESM.docx]

**Supplementary Table 1. Penetrance age for predisposition genes in prostate cancer (PC), breast cancer (BC), ovarian cancer (OvCa) and colorectal cancer (CRC).**

| Gene | Prevalence in PC (ref. 5) | Median age in PC | Median age in BC | Median age in OvCa | Median age in CRC | Reference for median age of BC, OvCa, CRC |
| --- | --- | --- | --- | --- | --- | --- |
| BRCA2 | 3.80% | 71 y | 40 y (US); 42y (Sweden) | 58 y |  | (1-3) |
| ATM | 2.70% | 60 y | x |  |  | (1) |
| CHEK2 | 2.50% | 63 y (1100delC) | x |  |  | (1) |
|  |  | 64 y (I157T) |  |  |  |  |
| BRCA1 | 0.80% | 66 y | 30s y (US); 50 y (Sweden) | 53 y |  | (1-3) |
| PALB2 | 0.40% |  | x |  |  | (1) |
| MSH2 | 1.20% | 65 y |  |  | 56 y | (4) |
| MLH1 | 0.10% | 70 y |  |  | 50 y | (4) |
| MSH6 | 0.80% |  |  |  | 62 y | (4) |
| PMS2 | 0.60% |  |  |  |  |  |
| HOXB13 | 1.20% | 62-63 y (G84E) |  |  |  |  |

According to Pritzlaff the prevalence of common pathogenic variants among PC predisposition genes decreased in order *BRCA2* 3.8%, *ATM* 2.7%, *CHEK2* 2.5%, *HOXB13* 1.2%, *MSH2* 1.2%, *BRCA1* 0.8%, *MSH6* 0.8%, *PMS2* 0.6% (**Supplementary Table 1**) (5). The results were reported by a Californian testing laboratory and several patients had diverse earlier cancers and/or family history of some cancers but the results are in line with other reports (6, 7). In PC, the median diagnostic age for *BRCA1* mutation carriers has been 66 years and that for *BRCA2* 71 years (8). For *ATM* mutation carriers median diagnostic age has been earlier, at 60 years (9). In hereditary PC, *CHEK2* 1100delC mutation carriers were found at a median age of 63 years compared to I157T carriers at age of 64 years (10). However in non-familial PC the diagnostic age has been 69 years (11). Enrichment of *ATM,* *CHEK2* and *PALB2* mutations have been reported in lethal PC (12, 13). According to the Prospective Lynch Syndrome Database cumulative incidence of PC by age 75 years was highest in *MSH2* mutation carriers (24%) and *MLH1* carriers (14%) with a median age of onset at 65 and 70 years, respectively (4). Mutations in *HOXB13* show extensive geographic variation with high frequency of risk alleles in Finland and Scandinavia (from 1 to 3% of PCs) (14-16). The median age of onset for the G84E carriers has been 62-63 years (14). However, lower ages were reported in US hereditary PC but also higher ages have been reported from UK (17, 18). The G84E variant is not associated with colorectal cancer while it may have a weak association with breast cancer (19).

It is noteworthy that the median diagnostic age for important cancer predisposition genes in PC differs extensively from that of other cancers (**Supplementary Table 1**). In US breast cancer patients, the median age of onset of *BRCA1* related mutations is in the 30s and that for *BRCA2* at around 40 years; however, early diagnostic age was not a feature of genes *ATM*, *CHEK2*, or *PALB2* (1). In Swedish breast cancer patients, the median onset is later, about 42 years for *BRCA1* and 50 years for *BRCA2* (3). In ovarian cancer the median age of diagnosis for *BRCA1* and *BRCA2* is at ages 53 and 58 years (2). In colorectal cancer mismatch repair gene mutations appeared far earlier than in PC, median ages being around 50 years for *MLH1*, 56 years for *MSH2* and 62 years for *MSH6* (4). For endometrial cancer the median ages were close to colorectal cancer, but for *MSH2* and *MSH6* lower, about 55 years (2).

1. Daly MB, Rosenthal E, Cummings S, Bernhisel R, Kidd J, Hughes E, et al. The association between age at breast cancer diagnosis and prevalence of pathogenic variants. Breast Cancer Res Treat. 2023;199(3):617-26.

2. Marchetti C, Ataseven B, Cassani C, Sassu CM, Congedo L, D'Indinosante M, et al. Ovarian cancer onset across different BRCA mutation types: a view to a more tailored approach for BRCA mutated patients. Int J Gynecol Cancer. 2023;33(2):257-62.

3. Winter C, Nilsson MP, Olsson E, George AM, Chen Y, Kvist A, et al. Targeted sequencing of BRCA1 and BRCA2 across a large unselected breast cancer cohort suggests that one-third of mutations are somatic. Ann Oncol. 2016;27(8):1532-8.

4. Dominguez-Valentin M, Sampson JR, Seppälä TT, Ten Broeke SW, Plazzer JP, Nakken S, et al. Cancer risks by gene, age, and gender in 6350 carriers of pathogenic mismatch repair variants: findings from the Prospective Lynch Syndrome Database. Genet Med. 2020;22(1):15-25.

5. Pritzlaff M, Tian Y, Reineke P, Stuenkel AJ, Allen K, Gutierrez S, et al. Diagnosing hereditary cancer predisposition in men with prostate cancer. Genet Med. 2020;22(9):1517-23.

6. Chung JH, Dewal N, Sokol E, Mathew P, Whitehead R, Millis SZ, et al. Prospective Comprehensive Genomic Profiling of Primary and Metastatic Prostate Tumors. JCO Precis Oncol. 2019;3.

7. Paulo P, Cardoso M, Brandão A, Pinto P, Falconi A, Pinheiro M, et al. Genetic landscape of homologous recombination repair genes in early-onset/familial prostate cancer patients. Genes Chromosomes Cancer. 2023;62(12):710-20.

8. Nyberg T, Frost D, Barrowdale D, Evans DG, Bancroft E, Adlard J, et al. Prostate Cancer Risks for Male BRCA1 and BRCA2 Mutation Carriers: A Prospective Cohort Study. Eur Urol. 2020;77(1):24-35.

9. Karlsson Q, Brook MN, Dadaev T, Wakerell S, Saunders EJ, Muir K, et al. Rare Germline Variants in ATM Predispose to Prostate Cancer: A PRACTICAL Consortium Study. European urology oncology. 2021;4(4):570-9.

10. Seppälä EH, Ikonen T, Mononen N, Autio V, Rökman A, Matikainen MP, et al. CHEK2 variants associate with hereditary prostate cancer. Br J Cancer. 2003;89(10):1966-70.

11. Wang Y, Dai B, Ye D. CHEK2 mutation and risk of prostate cancer: a systematic review and meta-analysis. Int J Clin Exp Med. 2015;8(9):15708-15.

12. Rantapero T, Wahlfors T, Kähler A, Hultman C, Lindberg J, Tammela TL, et al. Inherited DNA Repair Gene Mutations in Men with Lethal Prostate Cancer. Genes. 2020;11(3).

13. Wokołorczyk D, Kluźniak W, Stempa K, Rusak B, Huzarski T, Gronwald J, et al. PALB2 mutations and prostate cancer risk and survival. Br J Cancer. 2021;125(4):569-75.

14. Karlsson R, Aly M, Clements M, Zheng L, Adolfsson J, Xu J, et al. A population-based assessment of germline HOXB13 G84E mutation and prostate cancer risk. Eur Urol. 2014;65(1):169-76.

15. Storebjerg TM, Høyer S, Kirkegaard P, Bro F, Ørntoft TF, Borre M, et al. Prevalence of the HOXB13 G84E mutation in Danish men undergoing radical prostatectomy and its correlations with prostate cancer risk and aggressiveness. BJU Int. 2016;118(4):646-53.

16. Chen H, Ewing CM, Zheng S, Grindedaal EM, Cooney KA, Wiley K, et al. Genetic factors influencing prostate cancer risk in Norwegian men. Prostate. 2018;78(3):186-92.

17. Nyberg T, Govindasami K, Leslie G, Dadaev T, Bancroft E, Ni Raghallaigh H, et al. Homeobox B13 G84E Mutation and Prostate Cancer Risk. Eur Urol. 2019;75(5):834-45.

18. Ewing CM, Ray AM, Lange EM, Zuhlke KA, Robbins CM, Tembe WD, et al. Germline mutations in HOXB13 and prostate-cancer risk. N Engl J Med. 2012;366(2):141-9.

19. Laitinen VH, Wahlfors T, Saaristo L, Rantapero T, Pelttari LM, Kilpivaara O, et al. HOXB13 G84E mutation in Finland: population-based analysis of prostate, breast, and colorectal cancer risk. Cancer Epidemiol Biomarkers Prev. 2013;22(3):452-60.
